# Effects of van der Waals Interaction on N_2_ Adsorption on Carbon Nanotubes: Proposal for New Force Field Parameters

**DOI:** 10.1021/acsomega.5c12920

**Published:** 2026-03-06

**Authors:** Carlos Alberto Martins Junior, Henrique Musseli Cezar, Daniela Andrade Damasceno, Caetano Rodrigues Miranda

**Affiliations:** † 28133 Universidade de São Paulo, Instituto de Fisica, Rua do Matao 1371, São Paulo, São Paulo 05508-090, Brazil; ‡ Hylleraas Centre for Quantum Molecular Sciences and Department of Chemistry, 6305 University of Oslo, P.O. Box 1033 Blindern, 0315 Oslo, Norway

## Abstract

The separation of carbon dioxide (CO_2_) from nitrogen gas (N_2_) in flue gas has become an emerging strategy to mitigate climate change. Molecular simulations are valuable to provide insights for the gas separation process. A careful choice of force fields is required to avoid unrealistic predictions of thermodynamic properties. Most studies use Lorentz–Berthelot combining rules (LB) to obtain the interaction between different species. In this context, we verified how accurate LB is in describing the interaction of N_2_ molecules and carbon nanostructures by comparing the interaction energies of LB with those from density functional theory (DFT) calculations. Carbon nanomaterials were selected because they are considered promising materials to perform N_2_/CO_2_ separation. The results show that the LB underestimates the interaction energies and affects the prediction of the fundamental properties of solid–fluid interfacial interactions. To overcome this, we parametrized a Lennard–Jones potential using DFT and considering van der Waals interactions. The proposed potential shows good transferability and agreement with ab initio calculations. Molecular simulations were performed to verify the effects of employing LB in predicting the amount of nitrogen gas adsorbed in carbon nanotubes (CNTs). LB predicts a lower density within them. Our results suggest that LB leads to different adsorption properties.

## Graphical abstract

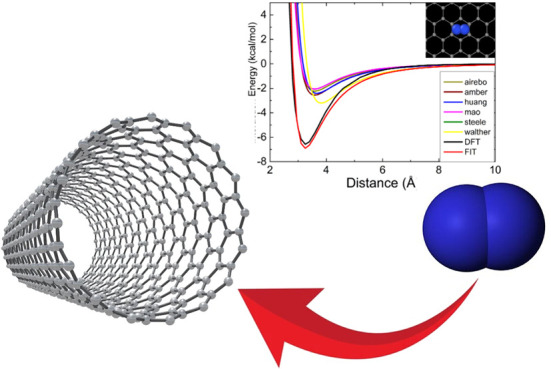

## Introduction

One of the most studied approaches to mitigate carbon dioxide (CO_2_) emissions is carbon capture and storage (CCS),^1^ which involves capturing greenhouse gases generated from fossil fuel combustion and storing them in geological formations. However, efficient separation of CO_2_ from the other flue gas components is a prerequisite for CCS. These gases are mainly composed of nitrogen (N_2_),^2,3^ which has driven numerous studies focused on improving the separation of this gas from carbon dioxide.^4^

A class of materials that exhibits promising properties for gas separation is carbon nanostructures.^5–7^ These materials are mechanically^8–10^ and chemically stable^11,12^ and offer tunable properties through functionalization, such as incorporating chemical radicals,^6^ or doping.^13^ For example, functionalized multiwalled carbon nanotubes have been shown to enhance the separation performance of mixed matrix membranes.^14^ Additionally, nanoporous graphene (NPG) has demonstrated the ability to selectively separate different gases,^15^ spurring further investigation into its separation properties. In the context of N_2_/CO_2_ separation, various pore diameters and geometries have been explored,^16,17^ along with the use of different atoms to passivate the nanopore rims.^16,18^ These studies have shown that NPGs are highly promising materials for N_2_/CO_2_ separation with selectivities reaching up to 100%.

To investigate gas separation mechanisms at the molecular scale, computational methods such as molecular dynamics (MD) and Monte Carlo (MC) simulations are commonly employed. In these simulations, interatomic interactions are typically described using classical force fields, most notably via the Lennard–Jones (LJ) potential, due to their low computational cost and ease of implementation. Cross-species interactions are generally estimated using the Lorentz–Berthelot (LB) combination rules: σ_*ij*_ = (σ_*ii*_ + σ_*jj*_)/2 and $\epsilon_{ij}=\sqrt{\epsilon_{ii}\epsilon_{jj}}$ , where indices *ii* and *jj* refer to the pure species, and *ij* to the interaction between them. Although widely adopted, these rules often fail to accurately capture interfacial interactions, particularly in systems dominated by van der Waals (vdW) forces.^7,20^ Machine learning interatomic potentials (MLIPs) have been proposed to address these shortcomings, offering quantum-level accuracy,^21^ but their application is still limited by the high cost of generating reference data sets and concerns about generalization in chemically diverse systems.^22,23^ In contrast, classical force fields remain the most practical choice for large-scale simulations, especially when exploring thermodynamic properties over a wide range of conditions.

Van der Waals interactions in carbon nanotube systems have also been extensively studied using continuum mechanics approaches, particularly in the context of multiwalled carbon nanotubes (MWCNTs). In these models, the Lennard–Jones potential is analytically integrated over continuous cylindrical surfaces to describe nanotube–nanotube or shell–shell interactions. Notably, Ru and co-workers proposed a formulation in which the interaction coefficient is assumed to be constant, while He and co-workers later introduced a radius-dependent interaction coefficient to improve the accuracy for nanotubes with different curvatures.^100^ These continuum formulations have been successfully applied to study the mechanical behavior, stability, and collapse of MWCNTs.^200^ However, they are not directly applicable to gas adsorption problems, where the relevant physics is governed by discrete atom–atom interactions between adsorbate molecules and the carbon framework.

In the specific case of N_2_ interacting with graphene, at least one force field surpasses the limitations of the LB rules: Vekeman et al. proposed new interaction parameters derived from density functional theory (DFT) calculations.^24^ However, the functional they employed, B97-D, is known to deviate significantly from the gold-standard CCSD­(T) method, which is considered to fully describe van der Waals interactions. For example, an 18.25% deviation was reported for coronene adsorbed on graphene using B97-D.^25^ More accurate results have been obtained using vdW-inclusive functionals such as KBM and C09.^26,27^ In particular, the adsorption energy of CO_2_ on graphene calculated with these functionals has shown good agreement with experimental values.^7,28^ As mentioned, for systems involving N_2_ (isolated molecules, dimers and clusters) and carbon nanomaterials, the vdW forces dominate the physisorption, while N_2_–N_2_ interactions are very weak, and vdW functionals should be preferred.^29^ Furthermore, Vekeman et al. conducted their DFT calculations using finite-sized molecular models to mimic graphene, which may introduce inaccuracies due to edge effects and the strategies used for border passivation.

In this work, we compared the adsorption energy profiles of N_2_ molecules on carbon structures obtained using various classical force fields with LB rules to those calculated with DFT using the KBM functional. As in our previous study,^7^ a significant discrepancy was observed between force field results and DFT, underscoring the need for a more accurate potential. We therefore developed a new set of cross parameters for the N–C that accurately reproduce the DFT adsorption energies of N_2_ on graphene and carbon nanotubes.

Since N_2_ is not only a major component of flue gases but also a standard probe molecule for characterizing porous materials, commonly used to derive surface area and pore size distributions, we further assessed the implications of underestimating vdW interactions on thermodynamic properties. For this, we performed Monte Carlo simulations to evaluate the N_2_ loading inside carbon nanotubes at 300 K and 1 atm, and constructed adsorption isotherms at 300 K, allowing for classification of isotherm types and analysis of the impact of interaction accuracy on gas uptake.

## Methods and Models

### Carbon Nanostructures

We considered the following carbon nanostructures: a 5 × 5 graphene sheet and (*m*, *n*) = (6, 6), (8, 8), (10, 10), and (0, 17) carbon nanotubes. These structures were created using the VMD software^30^ and the Topotools plug-in.^31^ For the N_2_ bond length, we used a value of 1.10 Å, while for the carbon nanomaterials, bond lengths were considered to be 1.418 Å.

### Classical Force Fields

To determine the accuracy of the LB combination rules in describing the interaction between N_2_ and carbon nanostructures, we compared the interaction energies obtained by using different classical FFs with those from DFT calculations.

In classical simulations, the most common way to describe van der Waals interactions is through the Lennard–Jones 12–6 potential, which is given by

1 $$E_{LJ}\left(r_{ij}\right)=4\varepsilon_{ij}\left[{\left(\frac{\sigma_{ij}}{r_{ij}}\right)}^{12}-{\left(\frac{\sigma_{ij}}{r_{ij}}\right)}^{6}\right]$$

where *ε*_*ij*_ is the depth of the potential well and σ_*ij*_ is the distance at which the interaction energy is zero.

For unlike atom pairs, the Lorentz–Berthelot combining rules are commonly used:

2 $$\sigma_{ij}=\frac{\sigma_{ii}+\sigma_{jj}}{2},,\varepsilon_{ij}=\sqrt{\varepsilon_{ii}\varepsilon_{jj}}$$

We used the AIREBO,^33^ Amber,^34^ Mao,^35^ Huang,^36^ Steele,^37^ and Walther^38^ force fields for the carbon structures and the Trappe model for N_2_. All these force fields use the Lennard–Jones potential for nonbonded interactions, with ϵ_*ii*_ and σ_*ii*_ presented in Table 1. We selected the Trappe model for N_2_ due to the small differences in the force field parameters of different models, as can also be seen in Table 1. The interaction energies were obtained at four different adsorption sites. The adsorption configurations are listed in Figure 1.

**Figure 1:**
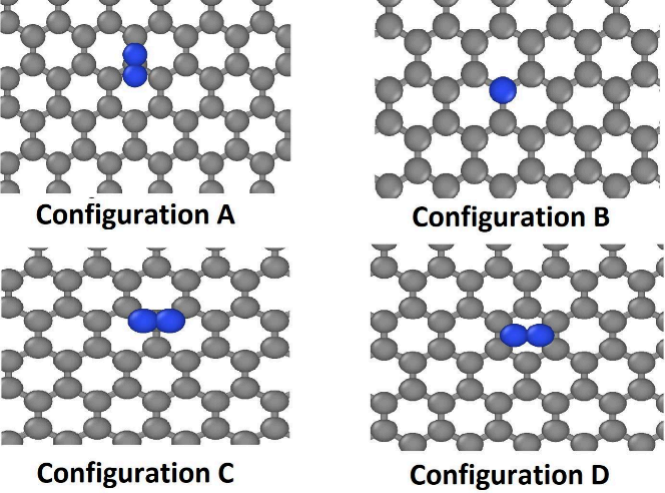
Schematic representation of the configurations used in the adsorption calculation. The gray and blue spheres represent the carbon and nitrogen atoms, respectively. In the figure, we used graphene as the carbon nanomaterial, but the same sites were also used for the carbon nanotubes. The visualizations were generated using OVITO.^32^

**Table 1:** Force Field Parameters for Carbon Structures (First Part of the Table) and Nitrogen Molecules (Second Part of the Table)^a^

force field	ϵ(*K*)	σ (Å)	*q*_*i*_ (*e*)
AIREBO^33^	32.96	3.400	-
Amber^34^	43.30	3.400	-
Mao^35^	48.78	3.370	-
Huang^36^	35.26	3.550	-
Steele^37^	28.00	3.400	-
Walther^38^	52.87	3.851	-
Trappe^39^	36.00	3.31	–0.482
Garcia-Perez^40^	36.40	3.32	–0.40484
Vujíc^41^	40.24	3.32	–0.482
Murthy^42^	36.44	3.33	–0.482

a All force fields for N_2_ studied here are three-site models. In such models, two sites are localized in each nitrogen atom, while the other is localized at the center of the molecule. The charge *q*_*i*_ is the value at the nitrogen atoms. For the site at the center of the molecule, the charge is – 2*q*_*i*_.

### DFT Calculations

Within the Kohn–Sham framework of density functional theory (DFT), the total energy of a system is written as

3 $$E_{DFT}\left[\rho \right]=T_{s}\left[\rho \right]+\int v_{ext}\left(r\right)\rho \left(r\right)dr+\frac{1}{2}\int \frac{\rho \left(r\right)\rho \left(r^{\prime }\right)}{\left|r-r^{\prime }\right|}dr\;dr^{\prime }+E_{xc}\left[\rho \right]$$

where *T*_s_[ρ] is the noninteracting kinetic energy, *v*_ext_ is the external potential due to the nuclei, the third term is the Hartree electrostatic energy, and *E*_xc_[ρ] is the exchange–correlation functional. In this work, a van der Waals inclusive exchange-correlation functional is employed, allowing long-range dispersion interactions to be explicitly incorporated.

The interaction energy is defined as

4 $$E_{int}=E_{sys}-E_{CNT}-E_{N_{2}}$$

where *E*_sys_ is the total energy of the combined system, and *E*_CNT_ and $E_{N_{2}}$ are the energies of the isolated subsystems.

DFT calculations were performed using the KBM exchange-correlation functional.^26^ This functional was found to successfully describe different systems in which the main interaction is due to van der Waals forces,^26,43^ as for N_2_ molecules interacting with carbon materials.^29^ Furthermore, recent studies have shown that the KBM functional gives good results for the adsorption energy of CO_2_ molecules on graphene, a system similar to the one investigated here.^7,28^ We also tested other vdW and non-vdW functionals. The vdW functionals tested were C09 ^27^ and BH,^44^ as well as an LDA functional, PW92,^45^ and a GGA functional, PBEsol.^46^ These tests did not show a significant difference between the vdW functionals, with the largest difference in the depth of the well being from KBM to C09, 0.4424 kcal/mol (7.2%). However, some significant differences were found from the vdW functionals to PW92 and PBEsol (see Figure S1 in the Supporting Information). Even though the LDA functional can describe the depth of the interaction energy, when compared to the vdW functionals, it cannot describe all the features of the interaction energies curves. These tests also show that GGA and PBEsol cannot describe either the depth of the interaction curve or the other parts. This pattern indicates the importance of including electron dispersion in the study of such systems.

All DFT calculations were performed using the SIESTA code.^47^ We used a mesh cutoff of 400 Ry for the grid and a convergence criterion of 1 × 10^–4^ in the elements of the density matrix. The selected basis was a double-ζ plus polarization orbitals, with the second zeta carrying 0.35 of the norm. Based on convergence tests, we selected an 8 × 8 × 1 k-points grid for the single-point calculations involving the 5 × 5 graphene sheet and 1 × 1 × 1 k-point for the CNTs. All DFT calculations considered periodic boundary conditions. Thus, to avoid interaction between the images of the system, the distance between the graphene sheets was 50 Å, and the CNTs were placed in the center of a 20 Å × 20 Å plane. We also tested the convergence of the interaction energy on the length of the carbon nanotubes, which is presented in Figure S2 of the Supporting Information. Based on these calculations, a length of 7.368 Å was selected for the arm-chair nanotubes and 8.508 Å for the zigzag nanotube.

### Classical Molecular Simulations

In classical molecular simulations, the nonbonded energy, which allows for adsorption phenomena, here, is decomposed as

5 $$E_{nonbonded}=\underset{i<j}{\sum }\left[E_{LJ}\left(r_{ij}\right)+E_{Coul}\left(r_{ij}\right)\right]$$

where *E*_Coul_ is the Coulomb interaction between partial charges and *E*_LJ_ is the Lennard–Jones potential.

In the specific case of N_2_ interacting with carbon nanostructures, carbon atoms carry no partial charges; therefore, the Coulomb contribution vanishes, and the adsorption energy is governed exclusively by the Lennard–Jones interaction.

In this study, the interaction energies for the classical force fields were obtained using LAMMPS,^48^ and the Moltemplate software^49^ was used to create the inputs. The energies were calculated using the compute group/group command, which does not include the interactions between the periodic images. Consequently, there was no need to test the convergence of the adsorption energy on the CNT length, as we did for the DFT calculations. Also, since we used classical force fields that use the Lennard–Jones potential, which is a pairwise potential, an effect due to many-body interactions in the cross-interactions between the periodic images does not exist, as might happen in the DFT calculations.

Grand Canonical Monte Carlo (GCMC) simulations were performed using the Cassandra software^50^ in order to obtain the N_2_ density inside different carbon nanotubes using the force fields tested here and the fitted potential. The chemical potential of N_2_ at 300.0 K and 1.0 atm, was obtained using the Widom insertion method.^51^ These simulations were carried for 4 × 10^6^ MC steps, with the last 3 × 10^6^ steps being considered for the production phase. Then, using this chemical potential, we performed GCMC simulations to load 200 Å long CNTs with N_2_. Four different CNTs were considered: (6,6), (8,8), (10,10) and (12,12). Initially, 5 × 1 × 10^4^ simulation steps in the NVT ensemble were performed to obtain the initial configuration. Then, a GCMC simulation of 5 × 10^6^ MC steps was performed, in which molecules were translated, rotated, inserted, or deleted inside the carbon nanotubes with a 25% probability for each move. For this last simulation, we employed configurationally biased insertions with 16 trial insertions. A cutoff of 15 Å was used for the Lennard–Jones plus Coulomb potential along with the Ewald method for long-range electrostatics, with the accuracy parameter set to 10^–5^.

To verify the effects of the different force fields on adsorption properties, we also used GCMC simulations to obtain adsorption isotherms of CNT (10,10) and (0,17) at *T* = 300 K, using the Steele–Waller and fitted force fields. We selected the Steele force field since it predicts the smallest density on CNT (10,10), as can be seen in the Results. For these simulations, we first simulated bulk N_2_ with different chemical potentials at the GCMC ensemble to obtain the average value of the pressure. Simulations of 3.5 × 10^6^ MC steps were performed to reach the equilibrium, and, then, 3 × 10^6^ MC steps were performed for the production phase. We used a tail correction for the simulations of the bulk, while the other simulation parameters were the same as previously discussed. Then, other simulations were performed to insert N_2_ molecules inside the carbon nanotubes. With these last simulations, we matched the number of adsorbed molecules inside the CNTs to the bulk pressure, obtaining the adsorption isotherm. Aside from the tail correction, we used the same simulation parameters. For the calculations of the gas density, we used the accessible volume, which was found by monitoring the position of the N atoms inside the CNTs.

### Fitting Procedure

Due to the difference between the energy curves from the DFT calculations and the LB rules (see the Results), we fitted a new set of Lennard–Jones parameters for the interactions between nitrogen and (10,10) and (0,17) CNTs interfacial interactions using GULP.^52^ To better fit the minimum of the energy curves, a weight based on the Boltzmann distribution was used,

6 $$w=e^{E-E_{\min}/kT}$$

in which *w* is the weight, *E*_min_ is the minimum of the curve, *k* is the Boltzmann constant, and *T* = 350 K is a temperature that is selected just to obtain a better description around the curve minimum. We also added another weight to the energies to balance the fact that the number of forces is greater since there are 3*N* forces components, *N* being the number of atoms, for each point in the interaction energy curve. The temperature and energy weight were selected by manually checking how well the fitting was compared to DFT energies and forces.

## Results and Discussion

### Comparison between DFT and FFs

Figure 2 shows the adsorption energies curves obtained with DFT, the classical force fields using LB combination rules, and the proposed potential. These results indicate that classical force fields underestimate the interaction energy between the nitrogen molecule and graphene or carbon nanotubes. Neither the position nor the depth of the potential wells is properly described. Since the parameters for both isolated systems can describe their interactions, we attribute this discrepancy to the LB combination rules.

**Figure 2:**
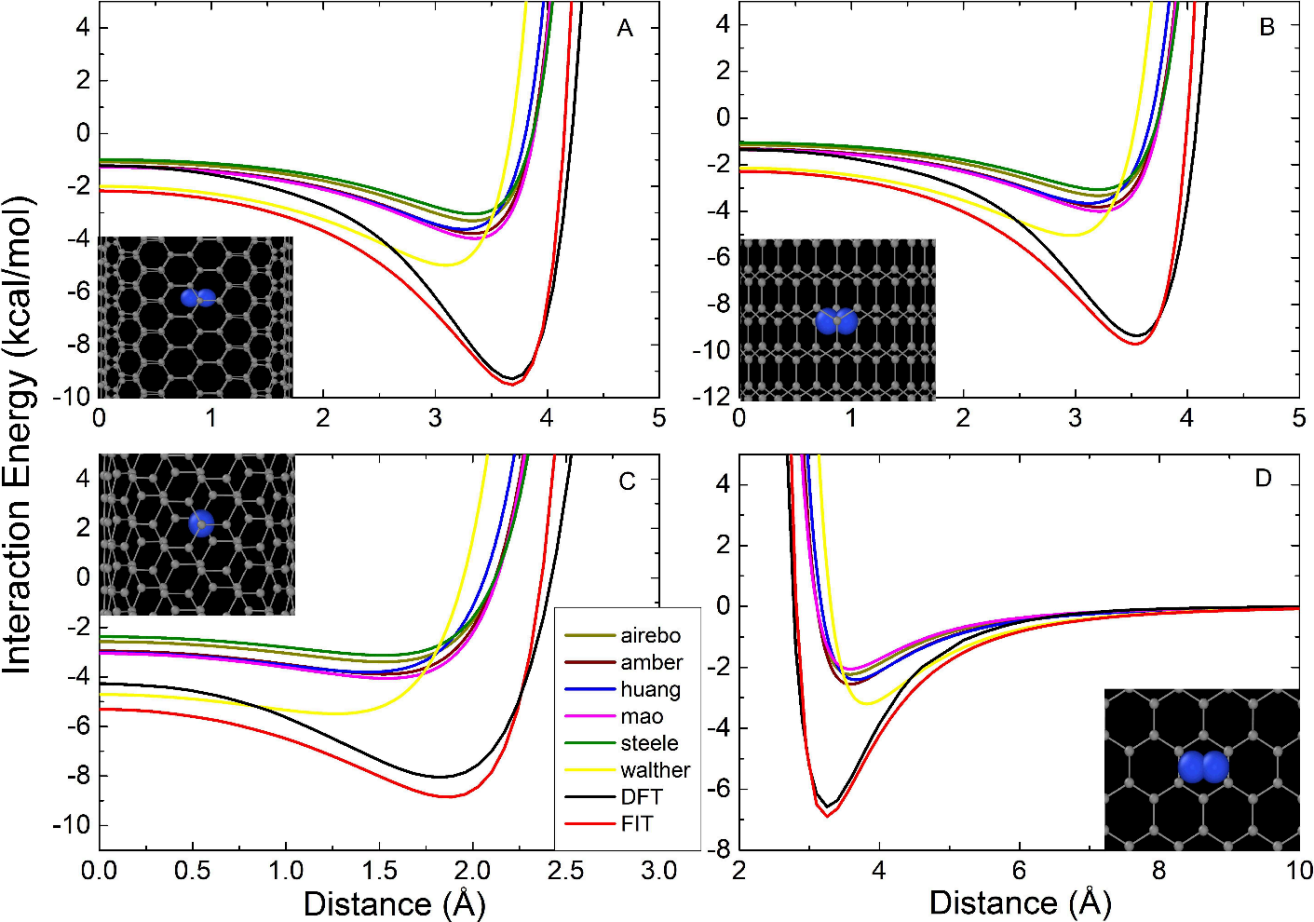
A) (10,10) CNT, B) (0,17) CNT, C) (8,8) CNT, and D) graphene. Comparison of the interaction energies of N_2_ and different carbon nanostructures, using DFT-KBM, fitted LJ parameters, and classical forces fields through Lorentz–Berthelot combination rules. See the text for details about the fitting procedure. The insets include the color-coded legend indicating whether the results come from DFT calculations or from the fitted potential.

The results also indicate that the adsorption site B (see Figure 1) is energetically the least favorable configuration, and aside from it, only slight differences between the other adsorption curves were observed, indicating no clear preferable adsorption site. A similar result was reported in the literature. Using molecular dynamics simulations, Vekeman et al. has recently shown that if the flexibility of the graphene sheet is taken into account, an united-atom model for nitrogen molecules gives almost the same results as an atomistic model.^53^ This suggests that the effect of the orientation of nitrogen molecules is negligible. Moreover, it is possible to conclude that vdW interaction does not play a role in the orientation since the results from KBM and their results, derived from the B97-D functional,^24^ which does not include electron dispersion, give the same result for nonpreferable orientation of N_2_ molecules on graphene.

Some discrepancies in the adsorption energies from our DFT calculations and others reported in the literature were found. We found an adsorption energy of −6.6 kcal/mol of N_2_ on graphene, while a value of −2.6 kcal/mol was reported using the B97-D functional,^24^ and a value of −3.2 kcal/mol was reported using ωB97X-D/cc-pVDZ.^54^ We attribute these differences to the different levels of calculation employed in each work. First, in the cited studies, periodic boundary conditions were not used, which might affect the adsorption energies since the finite size and strategies used to passivate the graphene border may affect the results. Second, it is known that the methodology employed in such studies leads to energies that are different from the ones obtained with coupled clusters calculations (CCSD­(T)), which fully describes van der Waals interactions.^55^ For example, a deviation of 18.25% between DFT-B97-D and CCSD­(T) was observed for coronene adsorbed on graphene.^25^ Furthermore, the KBM exchange-correlation functional employed here was found to successfully describe the adsorption energy of carbon dioxide on graphene.^7,28^

Unfortunately, to the best of our knowledge, there are no experimental data for N_2_ adsorption energies on graphene, as in the case of CO_2_. The only experimental results available are for the adsorption energy of N_2_ on graphite, in which values of −2.236 to −2.624 kcal/mol have been reported for the depth of the well.^56^ However, the adsorption energy on graphite can be significantly different from graphene.^7,28^

### Fitting

Due to the reported underestimation of the adsorption energies, we performed a fit of the Lennard–Jones cross parameters. During the fitting, we used forces and energies only from the (10,10) and (0,17) CNTs. The fitted LJ cross parameters are shown in Table 2. Figure 2 shows the curves for the interaction energies obtained from DFT, the fitted potential, and the classical forces fields employing the LB combination rules. The results show that the differences between the proposed FIT and the DFT calculations are within chemical accuracy, less than 1.0 kcal/mol. Using the fitted parameters to compute the interaction energy curves of other models, namely graphene and (8,8) CNT (Figure 2C and D), we observe that the FIT is transferable for smaller and larger curvatures.

**Table 2:** Fitted Lennard–Jones Parameters for N– C interactions

	ϵ_*ij*_ (K)	σ_*ij*_ (Å)
N–C	118.82	3.011

Figure 3 compares the forces acting on selected atoms by using the fitted parameters and DFT calculations. Overall, the curves are in reasonable agreement. The main force discrepancies occur when the nitrogen molecule is close to the carbon atoms in the nanostructure, as shown in Figure 3. As expected, at such short distances the interactions are energetically unfavorable, leading to a low probability of visiting those points in the phase space during a simulation. For example, at 2.5 Å from the center of the (8,8) nanotube, when the fit starts to diverge from the DFT reference (Figure 3A), the interaction energy is 2.56 kcal/mol, while the lowest energy in the same adsorption configuration is −8.02 kcal/mol. Despite describing the force on nitrogen atoms, the fit was unable to reproduce all of the features of the forces on carbon atoms. However, the difference is small and might not lead to distinct results in molecular dynamics simulations because of the thermal movement and the larger contribution of the bonded forces acting on such atoms. The oscillation present in DFT forces is due to the known eggbox effect present in our DFT calculations.^47^

**Figure 3:**
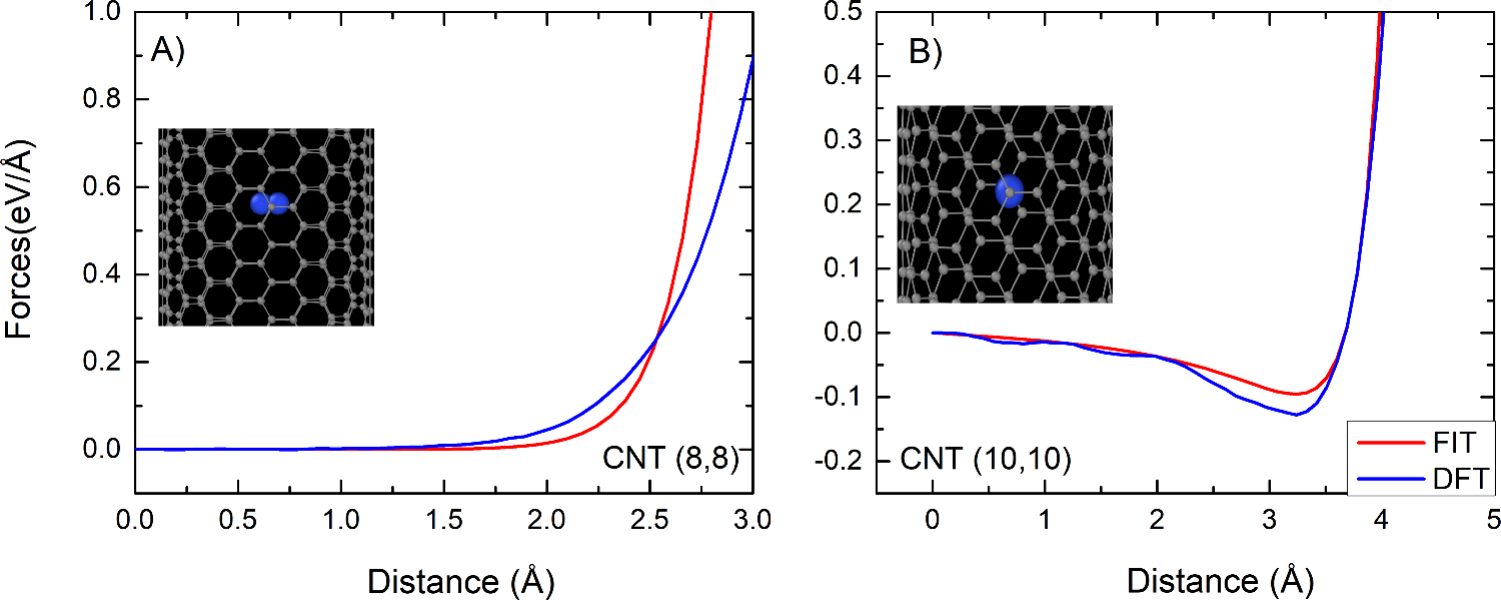
Comparison of the force component on N_2_ in the direction approaching the nanotube wall for A) (8,8) CNT and B) (10,10) CNT. The insets include the color-coded legend indicating if the results comes from the DFT calculations or from the fitted potential.

### Grand Canonical Monte Carlo Simulations

Figure 4 compares the density of nitrogen molecules inside different CNTs, using classical force fields with the LB and the proposed potential. Each FF predicts a different density, illustrating the importance of a careful selection of the most appropriate force field for molecular simulations. For all the nanotube diameters, our fit predicts a larger number of nitrogen molecules, reaching an almost 40 fold difference for the (12,12) CNT compared to Steele’s potential, respectively. The higher number of adsorbed molecules predicted by the fit can be related to the largest depth of the potential well of the carbon–nitrogen interaction and the σ, which is the distance where the potential is zero, being smaller. Because our fit agrees with the vdW DFT results, we suggest that the use of classical force fields and LB combination rules underestimates the vdW interaction between a nitrogen molecule and carbon nanotubes, predicting results that are different from those obtained with a good vdW description.

**Figure 4:**
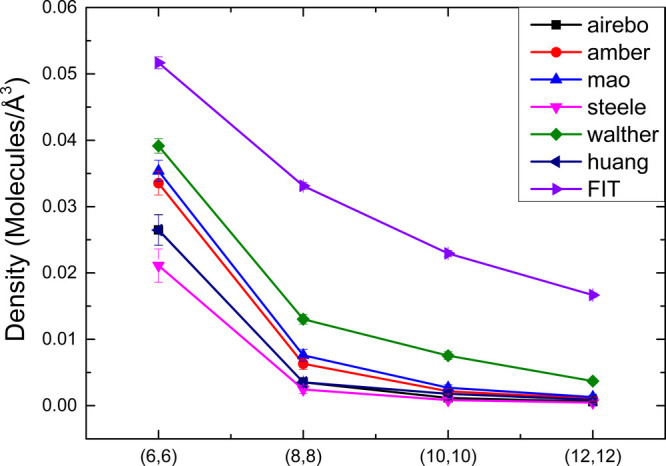
Comparison of the density of nitrogen molecules inside (6,6), (8,8), (10,10), and (12,12) carbon nanotubes. The proposed potential predicts a larger number of molecules inside the nanotubes in all cases.

Figure 5 shows the adsorption isotherms of the (10,10) and (0,17) CNTs, which have similar radii, obtained using the fitted parameters, and the Steele and Walther FFs using the LB rules. There is only a small difference between the isotherms of the two CNTs, indicating that chirality does not play an important role in the adsorption properties of CNTs when modeled with classical FFs. The difference is probably due to the slight differences in the volume of the CNTs.

**Figure 5:**
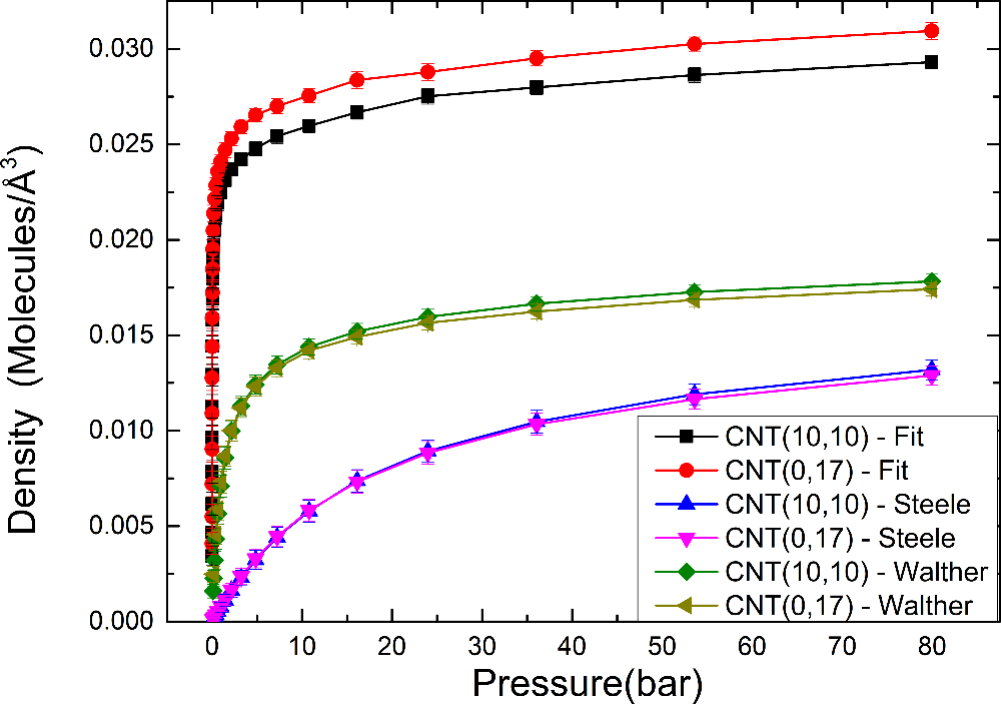
Adsorption isotherms of N_2_ in the (10,10) and (0,17) CNTs, predicted by Steele potential and LB and by the proposed potential. The *y*-axis is the number of adsorbed molecules inside the simulated CNTs, and this number is higher for the fitted potential, due to the larger potential depth.

Following the procedure described in Kumar et al.,^57^ each potential predicts a different isotherm curve, described by a different isotherm model. As can be seen in Figure S3, the isotherms predicted by the Steele potential are modeled by a Langmuir isotherm given by eq 7,

7 $$q_{h}=\frac{NKP}{1+KP}$$

in which *q*_*h*_ is the concentration of adsorbed molecules, *N* is the number of adsorption sites, *K* is related to the binding energy, and *P* is the pressure. The isotherm predicted from Walther’s potential is modeled by a combination of Langmuir equations, as can be seen in the plot of a Scatchard equation in Figure S4. Our fitted potential predicts that the isotherms are modeled by a Freundlich isotherm, as shown in Figure S5 of the Supporting Information, in agreement with experiments.^58–60^ The Freundlich model for adsorption isotherms is listed in eq 8.

8 $$q_{h}=q_{m}bP^{n}$$

The term *q*_m_ is the maximum adsorption capacity, and *b* is an isotherm constant.^57^

The reason for obtaining different isotherm models with each potential lies in the underestimation of the difference in adsorption energies in different adsorption sites. This is because a single Langmuir equation predicts only one adsorption energy, whereas the Freundlich isotherm predicts a distribution. Indeed, the difference in energy between configurations A and C predicted using the Steele potential is 0.002 kcal/mol, while the same difference using our fitted potential is 0.19 kcal/mol, both of which are below the thermal energy at room temperature (0.593 kcal/mol). Therefore, the results suggest that the underestimation of the adsorption energy from the LB combination rules also leads to an underestimation of the energy difference among the different adsorption sites, which changes the adsorption properties of a material.

## Conclusion

In this work, we investigated the adsorption of N_2_ in carbon nanostructures using a computational approach. We systematically compared the interaction energies using classical FFs within the LB rules, as often used in the literature, with those of DFT with the KBM vdW functional. We found that the force fields with LB underestimate the energies regardless of the potential used for carbon nanostructures. Due to this discrepancy, we fitted a new Lennard–Jones potential parameters for the N_2_–C interactions based on our DFT calculations. Our fitted potential shows much better accuracy for a range of CNTs and graphene, showing good transferability. We also compared adsorption isotherms and found that the classical FFs with LB tend to underestimate the load, causing the results obtained using this approximation to correspond to those of a lower temperature or lower pressure. The better accuracy of our potential opens up a more accurate interpretation of the favorable adsorption sites and a more accurate understanding of adsorption properties. Moreover, we also found that the type of isotherms also changes with our proposed potential, showing an isotherm that is fitted by the Freundlich model, instead of an Langmuir isotherm as in the other FFs with LB. All these differences in the adsorption isotherms are of great relevance because N_2_ is often used to measure the pore surface area of nanomaterials and because the atomistic view provided by molecular simulations is completely dependent on the description of the gas-framework interactions. We hope that the parametrization provided in this work opens up more accurate simulations with greater predictive power in the study of adsorption processes and separation studies. More generally and as importantly, these results also again expose the limitations of the LB rules in modeling solid–gas systems.

## Supplementary Material



## Notes

additional density functional theory (DFT) results assessing the role of van der Waals interactions on N_2_ adsorption on graphene and carbon nanotubes, including interaction energy curves obtained with different exchange–correlation functionals. Additional analyses addressing finite-size effects in periodic carbon nanotube models are provided. The Supporting Information also contains adsorption isotherm analyses based on Langmuir and Freundlich models, including linearized plots used to extract adsorption energies and characterize surface heterogeneity (PDF)

## #

(D.A.D.) University of São Paulo, Department of Mechatronics and Mechanical, Systems Engineering, Polytechnic School of the University of São Paulo, São Paulo 05508-030, Brazil

## References

[1] Gibbins J., Chalmers H. (2008). Carbon capture and storage. Energy
Policy.

[2] Granite E. J., Pennline H. W. (2002). Photochemical Removal of Mercury from Flue Gas. Energy Policy.

[3] Park, S.-E. ; Chang, J.-S. ; Lee, K.-W. Carbon Dioxide Utilization for Global Sustainability; Elsevier, 2003.

[4] Chawla M., Saulat H., Masood Khan M., Mahmood Khan M., Rafiq S., Cheng L., Iqbal T., Rasheed M. I., Farooq M. Z., Saeed M., Ahmad N. M., Khan
Niazi M. B., Saqib S., Jamil F., Mukhtar A., Muhammad N. (2020). Membranes for CO_2_ /CH_4_ and CO_2_/N_2_ Gas Separation. Chemical
Engineering and Technology.

[5] Sanip S. M., Ismail A. F., Goh P. S., Norrdin M., Soga T., Tanemura M., Yasuhiko H. (2011). Carbon Nanotubes Based Mixed Matrix
Membrane for Gas Separation. Advanced Materials
Research.

[6] Sanip S.M., Ismail A.F., Goh P.S., Soga T., Tanemura M., Yasuhiko H. (2011). Gas separation properties of functionalized carbon
nanotubes mixed matrix membranes. Sep. Purif.
Technol..

[7] Cezar H. M., Lanna T. D., Damasceno D. A., Kirch A., Miranda C. R. (2024). Revisiting
greenhouse gases adsorption in carbon nanostructures: Advances through
a combined first-principles and molecular simulation approach. Appl. Surf. Sci..

[8] Damasceno D. A., Hue K. Y., Miranda C. R., Müller E. A. (2025). Mechanical
Properties of Polyethylene/Carbon Nanotube Composites from Coarse-Grained
Simulations. Nanomaterials.

[9] Razmara N., Kirch A., Meneghini J. R., Miranda C. R. (2021). Efficient CH4/CO2
Gas Mixture Separation through Nanoporous Graphene Membrane Designs. Energies.

[10] Kirch A., Razmara N., Mamani V. F. S., Miranda C. R., Meneghini J. R. (2020). Multiscale
Molecular Modeling Applied to the Upstream Oil & Gas Industry
Challenges. Polytechnica.

[11] Yan L., Zheng Y. B., Zhao F., Li S., Gao X., Xu B., Weiss P. S., Zhao Y. (2012). Chemistry
and physics of a single
atomic layer: strategies and challenges for functionalization of graphene
and graphene-based materials. Chem. Soc. Rev..

[12] Galashev A. E., Rakhmanova O. R. (2014). Mechanical
and thermal stability of graphene and graphene-based
materials. Physics-Uspekhi.

[13] Lee H., Paeng K., Kim I. S. (2018). A review
of doping modulation in
graphene. Synth. Met..

[14] Lee R., Jawad Z., Ahmad A., Chua H. (2018). Incorporation of functionalized
multi-walled carbon nanotubes (MWCNTs) into cellulose acetate butyrate
(CAB) polymeric matrix to improve the CO2/N2 separation. Process Safety and Environmental Protection.

[15] Koenig S. P., Wang L., Pellegrino J., Bunch J. S. (2012). Selective molecular
sieving through porous graphene. Nat. Nanotechnol..

[16] Wang Y., Yang Q., Li J., Yang J., Zhong C. (2016). Exploration
of nanoporous graphene membranes for the separation of N2 from CO2:
a multi-scale computational study. Phys. Chem.
Chem. Phys..

[17] Wang S., Dai S., Jiang D.-e. (2019). Continuously
Tunable Pore Size for Gas Separation via
A Bilayer Nanoporous Graphene Membrane. ACS
Appl. Nano Mater..

[18] Liu H., Dai S., Jiang D.-e. (2013). Insights into CO2/N2 separation through nanoporous
graphene from molecular dynamics. Nanoscale.

[20] Delhommelle J., Millié P. (2001). Inadequacy
of the Lorentz-Berthelot combining rules
for accurate predictions of equilibrium properties by molecular simulation. Mol. Phys..

[21] Unke O. T., Chmiela S., Sauceda H. E., Gastegger M., Poltavsky I., Schütt K. T., Tkatchenko A., Müller K.-R. (2021). Machine Learning Force Fields. Chem. Rev..

[22] Wu S., Yang X., Zhao X., Li Z., Lu M., Xie X., Yan J. (2023). Applications and Advances in Machine Learning Force
Fields. J. Chem. Inf. Model..

[23] Poltavsky I. (2025). Crash testing machine
learning force fields for molecules, materials,
and interfaces: molecular dynamics in the TEA challenge 2023. Chemical Science.

[100] Ru C. (2000). Buckling of multiwalled carbon nanotubes
under radial pressure. Journal of the Mechanics
and Physics of Solids.

[200] He X., Lilley C. M. (2004). Radial deformation of double-walled carbon nanotubes
under compression. Journal of the Mechanics
and Physics of Solids.

[24] Vekeman J., Faginas-Lago N., Cuesta I. G., Sanchez-Marin J., De Meras A. S. (2018). Nitrogen Gas on Graphene: Pairwise Interaction Potentials. Computational Science and Its Applications - ICCSA 2018.

[25] Yeamin M. B., Faginas-Lago N., Alberti M., Cuesta I. G., Sanchez-Marin J., Sanchez de Meras A. M. J. (2014). Multi-scale theoretical investigation
of molecular hydrogen adsorption over graphene: coronene as a case
study. RSC Adv..

[26] Klimeš J., Bowler D. R., Michaelides A. (2010). Chemical accuracy
for the van der
Waals density functional. J. Phys.: Condens.
Matter.

[27] Cooper V. R. (2010). Van der
Waals density functional: An appropriate exchange functional. Phys. Rev. B.

[28] Takeuchi K., Yamamoto S., Hamamoto Y., Shiozawa Y., Tashima K., Fukidome H., Koitaya T., Mukai K., Yoshimoto S., Suemitsu M., Morikawa Y., Yoshinobu J., Matsuda I. (2017). Adsorption of CO2 on Graphene: A Combined TPD, XPS,
and vdW-DF Study. J. Phys. Chem. C.

[29] Shojaie F. (2018). N_2_ adsorption on the inside
and outside the single-walled carbon nanotubes
by density functional theory study. Pramana
- J. Phys..

[30] Humphrey W., Dalke A., Schulten K. (1996). VMD – Visual Molecular Dynamics. J. Mol. Graphics.

[31] Kohlmeyer, A. ; Vermaas, J. ; Braun, E. akohlmey/topotools: Release 1.8. 2020; https://zenodo.org/record/598373.

[32] Stukowski A. (2010). Visualization
and analysis of atomistic simulation data with OVITO-the Open Visualization
Tool. Modell. Simul. Mater. Sci. Eng..

[33] Brenner D. W., Shenderova O. A., Harrison J. A., Stuart S. J., Ni B., Sinnott S. B. (2002). A second-generation
reactive empirical bond order (REBO)
potential energy expression for hydrocarbons. J. Phys.: Condens. Matter.

[34] Cornell W. D., Cieplak P., Bayly C. I., Gould I. R., Merz K. M., Ferguson D. M., Spellmeyer D. C., Fox T., Caldwell J. W., Kollman P. A. (1995). A Second Generation Force Field for
the Simulation
of Proteins, Nucleic Acids, and Organic Molecules. J. Am. Chem. Soc..

[35] Mao Z., Garg A., Sinnott S. B. (1999). Molecular dynamics simulations of
the filling and decorating of carbon nanotubules. Nanotechnology.

[36] Huang L., Zhang L., Shao Q., Lu L., Lu X., Jiang S., Shen W. (2007). Simulations of Binary Mixture Adsorption
of Carbon Dioxide and Methane in Carbon Nanotubes: Temperature, Pressure,
and Pore Size Effects. J. Phys. Chem. C.

[37] Steele W. A. (1978). The interaction
of rare gas atoms with graphitized carbon black. J. Phys. Chem..

[38] Walther J. H., Jaffe R., Halicioglu T., Koumoutsakos P. (2001). Carbon Nanotubes
in Water: Structural Characteristics and Energetics. J. Phys. Chem. B.

[39] Potoff J. J., Siepmann J. I. (2001). Vapor–liquid equilibria of mixtures containing
alkanes, carbon dioxide, and nitrogen. AIChE
J..

[40] García-Pérez E., Parra J., Ania C., García-Sánchez A., van Baten J., Krishna R., Dubbeldam D., Calero S. (2007). A computational study of CO_2_, N_2_, and CH_4_ adsorption in zeolites. Adsorption.

[41] Vujić B., Lyubartsev A. P. (2016). Transferable force-field for modelling of CO2, N2,
O2 and Ar in all silica and Na+ exchanged zeolites. Modelling Simul. Mater..

[42] Murthy C., Singer K., Klein M., McDonald I. (1980). Pairwise additive effective
potentials for nitrogen. Mol. Phys..

[43] Hensley A. J. R., Ghale K., Rieg C., Dang T., Anderst E., Studt F., Campbell C. T., McEwen J.-S., Xu Y. (2017). A DFT-Based
Method for More Accurate Adsorption Energies: An Adaptive Sum of Energies
from RPBE and vdW Density Functionals. J. Phys.
Chem. C.

[44] Berland K., Hyldgaard P. (2014). Exchange functional that tests the robustness of the
plasmon description of the van der Waals density functional. Phys. Rev. B.

[45] Perdew J. P., Wang Y. (1992). Accurate and simple analytic representation of the electron-gas correlation
energy. Phys. Rev. B.

[46] Perdew J. P., Ruzsinszky A., Csonka G. I., Vydrov O. A., Scuseria G. E., Constantin L. A., Zhou X., Burke K. (2008). Restoring the Density-Gradient
Expansion for Exchange in Solids and Surfaces. Phys. Rev. Lett..

[47] Artacho E., Anglada E., Diéguez O., Gale J. D., García A., Junquera J., Martin R. M., Ordejón P., Pruneda J. M., Sánchez-Portal D., Soler J. M. (2008). The SIESTA
method: developments and applicability. J. Phys.:
Condens. Matter.

[48] Thompson A. P., Aktulga H. M., Berger R., Bolintineanu D. S., Brown W. M., Crozier P. S., in ’t Veld P. J., Kohlmeyer A., Moore S. G., Nguyen T. D., Shan R., Stevens M. J., Tranchida J., Trott C., Plimpton S. J. (2022). LAMMPS
- a flexible simulation tool for particle-based materials modeling
at the atomic, meso, and continuum scales. Comput.
Phys. Commun..

[49] Jewett A. I., Stelter D., Lambert J., Saladi S. M., Roscioni O. M., Ricci M., Autin L., Maritan M., Bashusqeh S. M., Keyes T., Dame R. T., Shea J.-E., Jensen G. J., Goodsell D. S. (2021). Moltemplate: A Tool for Coarse-Grained Modeling of
Complex Biological Matter and Soft Condensed Matter Physics. J. Mol. Biol..

[50] Shah J. K., Marin-Rimoldi E., Mullen R. G., Keene B. P., Khan S., Paluch A. S., Rai N., Romanielo L. L., Rosch T. W., Yoo B., Maginn E. J. (2017). Cassandra: An open
source Monte Carlo package for molecular simulation. J. Comput. Chem..

[51] Widom B. (1963). Some Topics
in the Theory of Fluids. J. Chem. Phys..

[52] Gale J. D. (1997). GULP: A
computer program for the symmetry-adapted simulation of solids. J. Chem. Soc., Faraday Trans..

[53] Vekeman J., Sanchez-Marin J., Sanchez de Meras A., Garcia Cuesta I., Faginas-Lago N. (2019). Flexibility
in the Graphene Sheet: The Influence on
Gas Adsorption from Molecular Dynamics Studies. J. Phys. Chem. C.

[54] Gordeev E. G., Polynski M. V., Ananikov V. P. (2013). Fast and accurate
computational modeling
of adsorption on graphene: a dispersion interaction challenge. Phys. Chem. Chem. Phys..

[55] Klimeš J., Michaelides A. (2012). Perspective:
Advances and challenges in treating van
der Waals dispersion forces in density functional theory. J. Chem. Phys..

[56] Vidali G., Ihm G., Kim H.-Y., Cole M. W. (1991). Potentials of physical adsorption. Surf. Sci. Rep..

[57] Kumar K. V., Gadipelli S., Wood B., Ramisetty K. A., Stewart A. A., Howard C. A., Brett D. J. L., Rodriguez-Reinoso F. (2019). Characterization
of the adsorption site energies and heterogeneous surfaces of porous
materials. J. Mater. Chem. A.

[58] Khalili S., Ghoreyshi A. A., Jahanshahi M., Davoodi M. (2013). Experimental Evaluation
of CO_2_ /N_2_ Mixture Separation by Multi-multi-walled
Carbon Nanotube. Acta Phys. Polym., A.

[59] Rahimi M., Singh J. K., Babu D. J., Schneider J. J., Müller-Plathe F. (2013). Understanding Carbon
Dioxide Adsorption in Carbon Nanotube
Arrays: Molecular Simulation and Adsorption Measurements. J. Phys. Chem. C.

[60] Erdogan F. O. (2019). Freundlich,
Langmuir, Temkin and Harkins-Jura isotherms studies of H_2_ adsorption on porous adsorbents. Chem. Chem.
Technol. (JCCT).

