# Supplementary material for: Effects of van der Waals Interaction on N2 Adsorption on Carbon Nanotubes: Proposal for New Force Field Parameters
Source: ACS Omega. 2026 Mar 6;11(11):18080–7. doi: 10.1021/acsomega.5c12920 (PMC13019377; doi:10.1021/acsomega.5c12920)
Supplement: Supplementary file 1 [file ao5c12920_si_001.pdf]

Supporting Information: Effects of van der Waals interaction on the N2 adsorption on carbon nanotubes: proposal of new force field parameters

Carlos Alberto Martins Junior,[*,†] Henrique Musseli Cezar,[*,†,‡] Daniela Andrade Damasceno ,[a*,†] and Caetano Rodrigues Miranda[*,†]

†*Universidade de Sao Paulo, Instituto de Fisica, Rua do Matao 1371, Sao Paulo, SP, 05508-090, Brazil*

‡*Hylleraas Centre for Quantum Molecular Sciences and Department of Chemistry, University of Oslo, PO Box 1033 Blindern, 0315 Oslo, Norway*

E-mail: camjjr.cm@usp.br; h.m.cezar@kjemi.uio.no; daniela.damasceno@usp.br; crmiranda@usp.br

[a]D.A.D. current adress is University of São Paulo, Department of Mechatronics and Mechanical, Systems Engineering, Polytechnic School of the University of São Paulo, São Paulo 05508-030, SP, Brazil

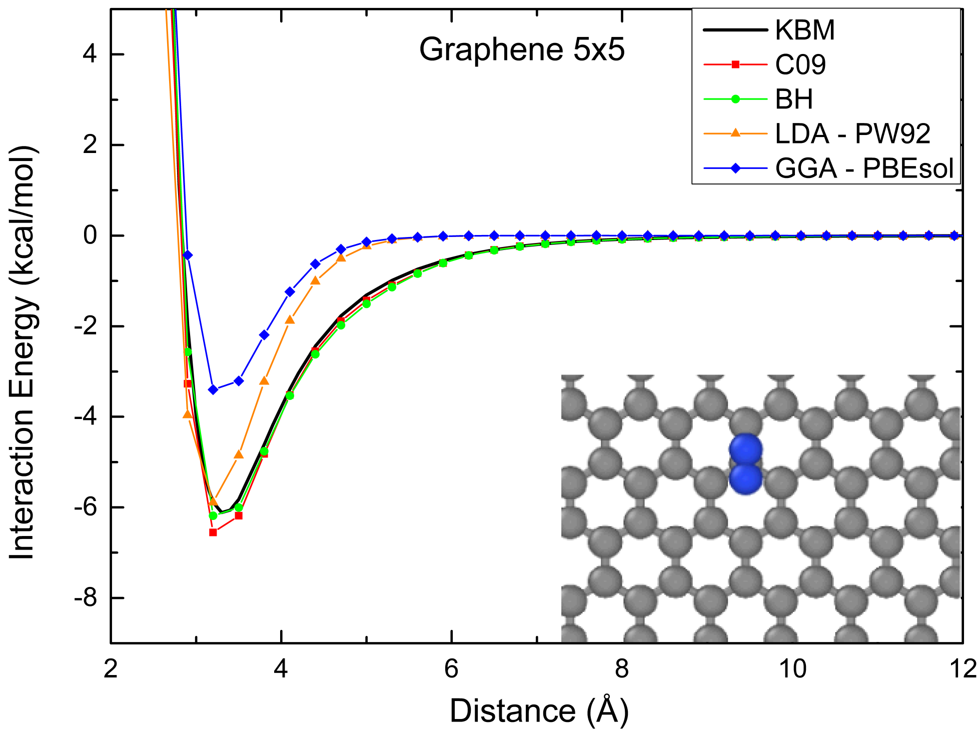

Figure S1: Interaction energy curve of a nitrogen molecule and graphene obtained from different functionals. All the van der Waals functionals agree with themselves with only small deviations. However, the results from LDA and GGA functional deviate significantly from those with Van der Waals functionals.

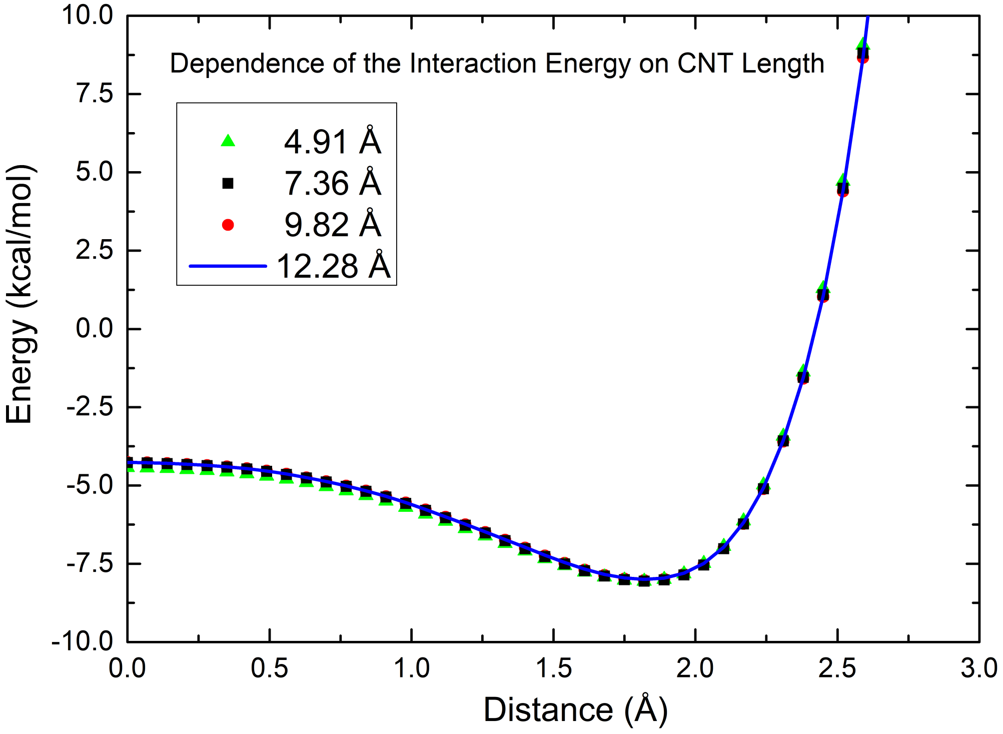

Figure S2: Interaction energy of a nitrogen molecule inside (10,10) CNTs with different lengths. Since our DFT calculations use periodic boundary conditions, the interaction between the images could affect the final results. However, as shown in the figure, the difference between the interaction energies obtained using the 7.36 Å CNT and the longer CNTs are negligible.

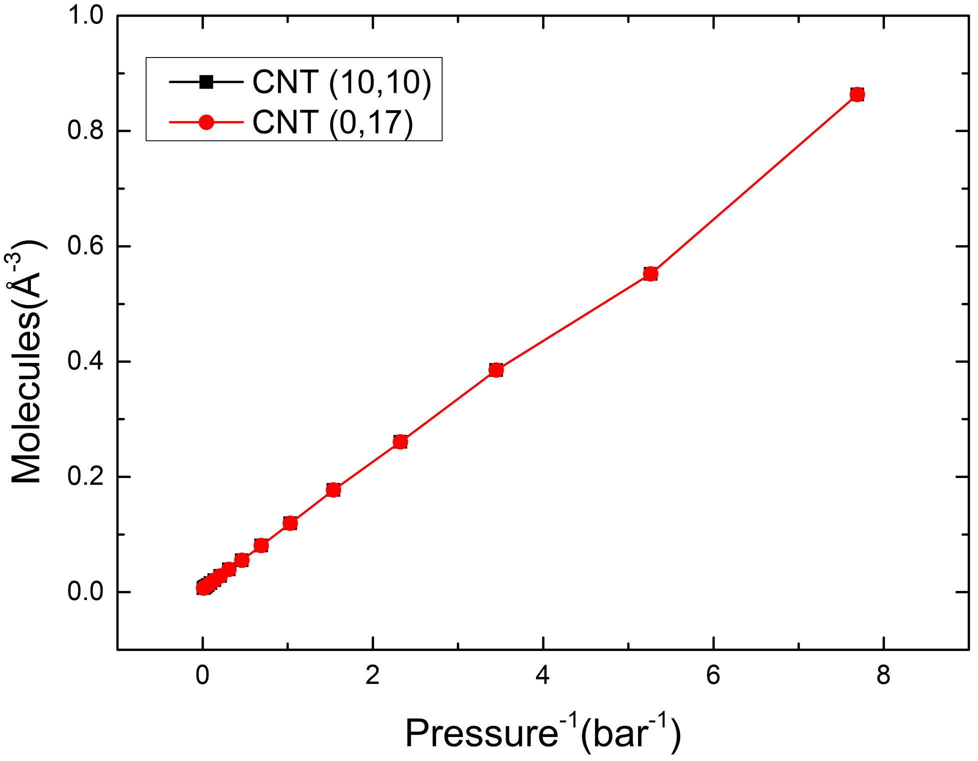

Figure S3: Plot of 1/Number of Molecules against 1/Pressure, which should give a straight line according to the Languir equation for isotherms.[S1]

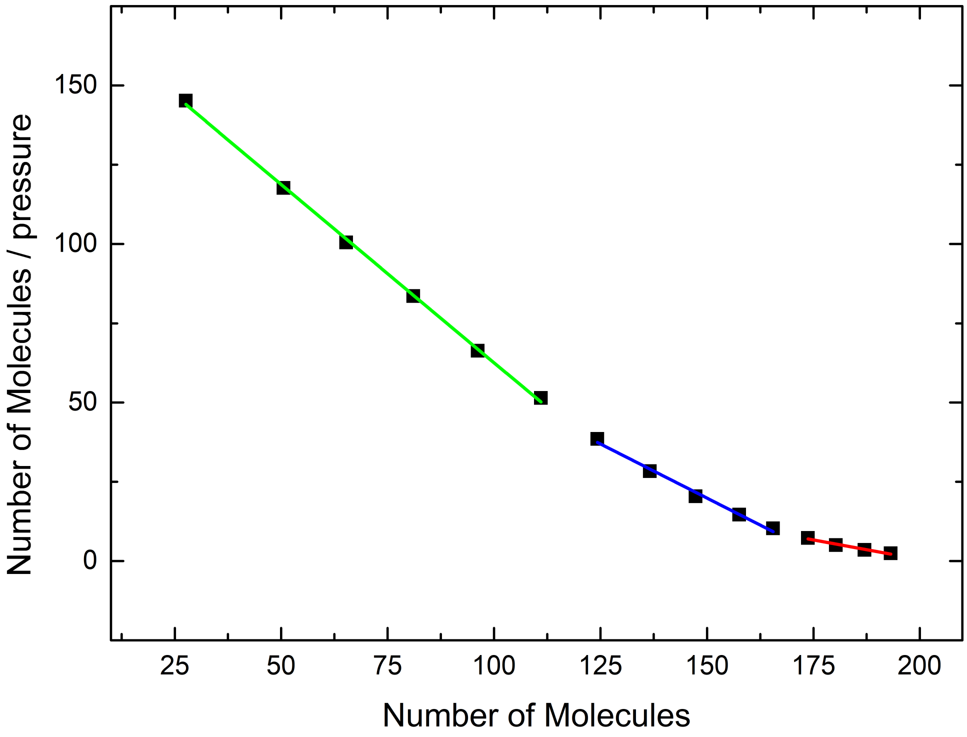

Figure S4: Plot of Number of Molecules/*Pressure* against Number of Molecules. The number of straight lines gives the number of adsorption energies.[S1]

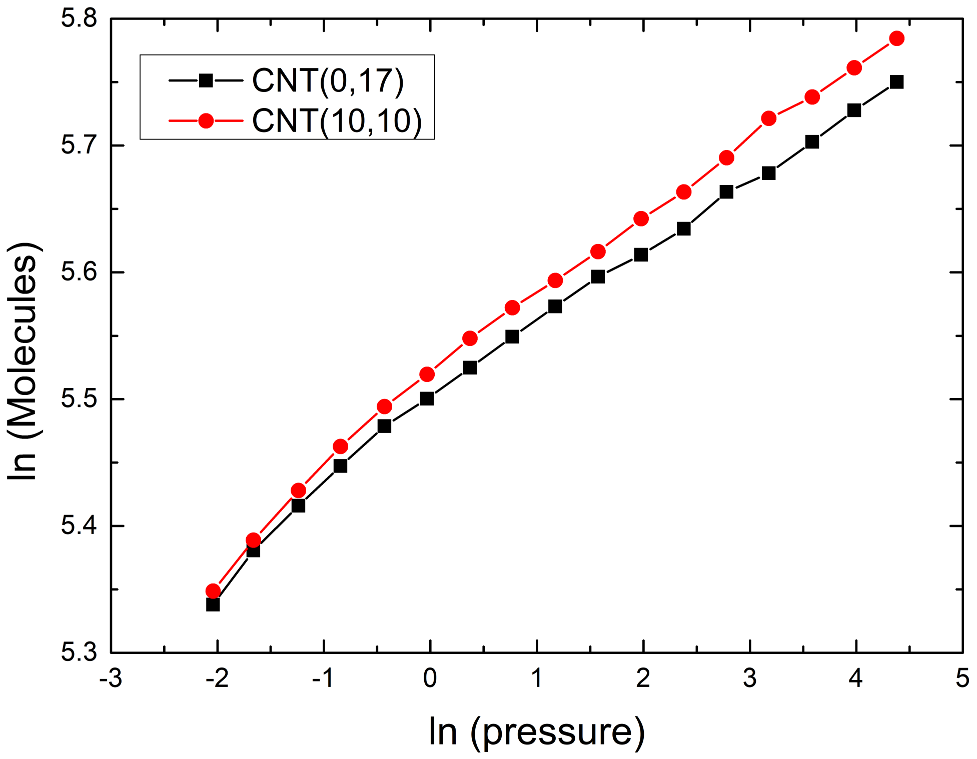

Figure S5: Plot of the natural logarithm of the Number of Molecules against the natural logarithm of Pressure, which should give a straight line according to the Freundlich model for isotherms.[S1]

References

(S1) Kumar, K. V.; Gadipelli, S.; Wood, B.; Ramisetty, K. A.; Stewart, A. A.; Howard, C. A.; Brett, D. J. L.; Rodriguez-Reinoso, F. Characterization of the adsorption site energies and heterogeneous surfaces of porous materials. *J. Mater. Chem. A* **2019**, *7*, 10104–10137.
